# Serum 25(OH)D Is a 2-Year Predictor of All-Cause Mortality, Cardiac Death and Sudden Cardiac Death in Chest Pain Patients from Northern Argentina

**DOI:** 10.1371/journal.pone.0043228

**Published:** 2012-09-06

**Authors:** Patrycja A. Naesgaard, Ricardo A. León De La Fuente, Stein Tore Nilsen, Leik Woie, Torbjoern Aarsland, Cato Brede, Harry Staines, Dennis W. T. Nilsen

**Affiliations:** 1 Department of Cardiology, Stavanger University Hospital, Stavanger, Norway; 2 Institute of Medicine, University of Bergen, Bergen, Norway; 3 Cardiology Research Institute, Catholic University of Salta, Salta, Argentina; 4 Department of Research, Stavanger University Hospital, Stavanger, Norway; 5 Department of Medical Biochemistry, Stavanger University Hospital, Stavanger, Norway; 6 Sigma Statistical Services, Balmullo, United Kingdom; University of Florida, United States of America

## Abstract

**Background:**

Several studies have shown an association between vitamin D deficiency and cardiovascular risk. Vitamin D status is assessed by determination of 25-hydroxyvitamin D [25(OH)D] in serum.

**Methods:**

We assessed the prognostic utility of 25(OH)D in 982 chest-pain patients with suspected acute coronary syndrome (ACS) from Salta, Northern Argentina. 2-year follow-up data including all-cause mortality, cardiac death and sudden cardiac death were analyzed in quartiles of 25(OH)D, applying univariate and multivariate analysis.

**Results:**

There were statistically significant changes in seasonal 25(OH)D levels. At follow-up, 119 patients had died. The mean 25(OH)D levels were significantly lower among patients dying than in long-term survivors, both in the total population and in patients with a troponin T (TnT) release (n = 388). When comparing 25(OH)D in the highest quartile to the lowest quartile in a multivariable Cox regression model for all-cause mortality, the hazard ratio (HR) for cardiac death and sudden cardiac death in the total population was 0.37 (95% CI, 0.19–0.73), p = 0.004, 0.23 (95% CI, 0.08–0.67), p = 0.007, and 0.32 (95% CI, 0.11–0.94), p = 0.038, respectively. In patients with TnT release, the respective HR was 0.24 (95% CI, 0.10–0.54), p = 0.001, 0.18 (95% CI, 0.05–0.60), p = 0.006 and 0.25 (95% CI, 0.07–0.89), p = 0.033. 25(OH)D had no prognostic value in patients with no TnT release.

**Conclusion:**

Vitamin D was shown to be a useful biomarker for prediction of mortality when obtained at admission in chest pain patients with suspected ACS.

**Trial registration:**

ClinicalTrials.gov NCT01377402

## Introduction

It is well known that Vitamin D deficiency in humans is widespread and increasing [1]. Vitamin D can be ingested or created in the skin on exposure to sun and is essentially derived from the latter source. Vitamin D status is commonly assessed by determination of 25-hydroxyvitamin D [25(OH)D] in serum [2].

Optimal and exact cut-off levels of 25(OH)D are still under debate. The following cut-off levels have been recognized; normal values 75–250 nmol/L, insufficiency 50–74 nmol/L and deficiency <50 nmol/L [3]–[4]. However, these values are based on registry data which do not fully take into account population and geographical differences, and factors such as gender and genetics [1].

Several observational studies and epidemiological data suggest that low levels of 25(OH)D may be related to mortality and cardiovascular disease (CVD) [5]–[12] such as myocardial infarction (MI) [13]–[14] and sudden cardiac death (SCD) [15].

The general diet does not contain a sufficient amount of vitamin D and without supplementation we depend on sun exposure to obtain a satisfactory level of this vitamin. The cutaneous synthesis of vitamin D is influenced by several factors, including geographical location, latitude, altitude, season and daytime, the color of the skin, age and obesity [3], [16]–[17].

Fish, the main diet source of vitamin D, is less preferred by the inland and highland beef-consuming population in Northern Argentina, resulting in a lower dietary intake of vitamin D. In our study population from Salta, Argentina, dietary insufficiency of vitamin D may be outweighed by the increased sun exposure through the entire year at this latitude (24 degrees south of the Equator) and an altitude above 1000 m. Therefore, we have assumed that sun exposure may be the essential energy source for the production of vitamin D synthesis in this population.

Thus, the purpose of this study was to assess the prognostic utility of 25(OH)D in 982 chest-pain patients with suspected acute coronary syndrome (ACS) living within the described area of Argentina.

## Methods

### Ethics Statement

The study was approved by the Ethics Committee at the Board of Medical School of Salta and conducted in accordance with the Helsinki Declaration of 1971, as revised in 1983. At San Bernardo Hospital and Sanatorio El Carmen, the study was also approved by the local Ethics Committee and Institutional Review Board of San Bernardo Hospital and the Institutional Review Board of Sanatorio El Carmen, respectively. The Norwegian biobank containing Argentinean blood samples was approved by the Regional Board of Research Ethics and the Norwegian health authorities. This study was monitored by Stavanger Health Research, Stavanger, Norway. Written informed consent was obtained from all patients.

### Study Design and Patient Population

This study was performed at nine hospitals in the Province of Salta, Northern Argentina and was given the acronym ARRA-RACS (ARgentinean Risk Assessment Registry in the Acute Coronary Syndrome), registered in ClinicalTrials.gov Identifier NCT01377402. It was designed to evaluate the prognostic utility of serum 25(OH)D (D represents D_2_ and D_3_) status in 982 patients, hospitalized consecutively with chest pain and a suspected ACS, from December 2005 to January 2009. The patients had to be above 18 years of age and willing to participate in this study after informed consent. Eight centers were private and one was public. The latter included sixty-two (6.5%) of the patient population.

The primary outcome was 2-year all-cause mortality from the time of inclusion and the secondary outcomes included cardiac death and sudden cardiac death.

During the two year follow-up period, the participants were interviewed by telephone at 30 days, 6 and 24 months, and they were invited for a physical examination at 12 months. Family, neighbours and the national registry department were contacted to obtain relevant information regarding relocations. Information related to cause of death was obtained through official records and additional information was supplied by close family members.

Clinical parameters based on hospital records and personal interviews included age, gender, assessment of previous MI, angina pectoris, previous revascularizations [percutaneous coronary intervention (PCI) or coronary artery bypass graft (CABG)], congestive heart failure (CHF) according to Killip-Kimball class [18], diabetes mellitus (DM), smoking status (categorized as current smokers, previous smokers or never-smokers), hypercholesterolemia (defined as total cholesterol concentrations above 250 mg/dl or statin treatment), beta-blocker use, arterial hypertension (defined as repeated blood pressure measurements above 140/90 mmHg or treated hypertension), body mass index (BMI) (kg/m^2^) and month of sample collection. Electrocardiographic findings at admission were classified according to the presence of ST-segment changes (i.e. ST-segment depression or elevation, T-wave inversion or left bundle-branch block).

Blood samples for determination of laboratory parameters were obtained at admission. A second blood sample for repeated troponin T (TnT) determination was drawn 6 hours following the primary blood sample. Baseline data included 25(OH)D_2_ and 25(OH)D_3_, TnT, high sensitivity C-reactive protein (hsCRP), glucose, serum lipids, B-type natriuretic peptide (BNP) measured in EDTA (ethylene diamine tetraacetic acid) plasma, and estimated glomerular filtration rate (eGFR), [calculated by Modification in Diet in Renal Disease (MDRD) formula].

The term ACS in this study included unstable angina pectoris, non ST-segment elevation myocardial infarction (NSTEMI) and ST-segment elevation myocardial infarction (STEMI), as previously described [19].

The definition of cardiac death included death preceded by a definitive MI or by chest pain >20 minutes without a given TnT, or a history of ischemic heart disease and no other obvious cause of death [20]. SCD is defined as unexpected death due to a cardiac cause occurring within one hour of symptom onset or as a witnessed unexpected death [21].

Furthermore, the total patient population was divided into two subgroups, with and without TnT release, respectively.

### Blood Sampling Procedures and Laboratory Measurements

Blood samples were drawn immediately following admission in a nonfasting state by direct venipuncture of an antecubital vein, applying minimum stasis. Clotted whole blood and EDTA blood samples were centrifuged for 15 min with 2000× g at 20°C without delay. Serum and EDTA plasma were frozen in three aliquots, stored locally at −70°C and transferred in frozen condition (dry ice) to Stavanger, Norway in three different shipments; after collection of the first 100 samples, the next 400 samples and finally the remaining samples, respectively. These samples were stored in the Norwegian biobank at −70°C.

Assessment of vitamin D status was performed by determination of the metabolites 25(OH)D_3_ and 25(OH)D_2_ in serum by liquid-liquid extraction (LLE), derivatization with 4-phenyl-1,2,4-triazoline-3,5-dione reagent (PTAD, Sigma-Aldrich, St. Louis, MO, USA), and analysis by liquid chromatography coupled with tandem mass spectrometry detection (LC-MS/MS). A one-step LLE procedure was performed by mixing 50 µl serum, 50 µl internal standard solution, comprising 160 ng/ml of 6-deuterium labeled 25(OH)D_3_ (Synthetica, Oslo, Norway) in isopropanol, 350 µl of 200 mmol/L magnesium sulphate, and finally 900 µl acetone and heptane (1+1). The upper heptane layer was acquired and evaporated, followed by addition of 100 µl of 0.5 mg/ml PTAD reagent in dry acetonitrile. The LC-MS/MS analysis was performed with an Acquity UPLC coupled with a Quattro Micro (Waters, Milford Massachusetts, USA). The separation was isocratic, using a 2.1×50 mm Acquity BEH C18 UPLC column (Waters) and a 0.5 mL/min mobile phase flow consisting of 20% ammonium hydroxide (0.1%) and 80% acetonitrile. Tandem mass spectrometry detection was with positive electrospray ionization (ESI+), using 3 kV and 30 V for capillary and cone voltage, respectively. Collision energies were 15 for 25(OH)D_3_ and 17 for 25(OH)D_2_. The multiple reaction monitoring transitions were 558.5>298.2 for 25(OH)D_3_, 564.5>298.2 for deuterium labeled 25(OH)D_3_, and 570.5>298.2 for 25(OH)D_2_.

Calibration was achieved by using serum calibrator #38033 (Chromsystems, Munich, Germany). With each microwell plate, the analytical quality was monitored by analysis of 5 different control samples: #0029 and #0030 (Chromsystems), #35080 and #35081 (Recipe, Munich, Germany), and HK10 (DEKS, Herlev, Denmark). The coefficients of variation (CV) for the control samples analyzed over 25 series were in the range of 8.7–10.8% for 25(OH)D_3_ and in the range of 10.7–16.5% for 25(OH)D_2_. Intra-series repeatability was estimated at three different levels, and CV's found in the range of 2.9–8.2%. Method bias was estimated by relative difference from the quality control sample values. For 25(OH)D_3_, 21–22% bias was found by analysis of control samples with a reference value of 38.6 and 59 nmol/L, and 1–2% bias was found for samples with a reference value of 73.4 and 136 nmol/L. For a control sample with a high reference value of 265 nmol/L, the bias was −18%. For 25(OH)D_2_, control samples with reference values of 39, 62 and 126 nmol/L, respectively, were associated with a bias in the range of −3 to 8%, and −9% for a sample with a high reference value of 252 nmol/L.

TnT was quantified by a cardiac-specific second-generation TnT ELISA assay from Roche diagnostics, using a high-affinity cardiac-specific TnT isoform antibody [22]. The lower detection limit of the assay used is 0.01 ng/mL. In this study a cut-off level of 0.05 ng/mL was used with a CV of 10% for the diagnosis of a MI, whereas patient groups in this study were defined according to TnT release (TnT>0.01 ng/ml).

BNP (Microparticle Enzyme Immunoassay Abbott AxSYM® (Abbott Laboratories, Abbott Park, Illinois, USA) and hsCRP [Tina-quant® C-reactive protein (latex) high sensitive assay, Roche Diagnostics, Germany] were analyzed as recommended by the manufacturer, and as previously described [19].

### Statistical Analysis

Patients were divided into quartiles according to their 25(OH)D levels. Approximately normally distributed variables were given as mean and standard deviation (SD). The Chi-square test for association was applied between the 25(OH)D quartiles and categorical variables at baseline. The one way ANOVA was used to test for equality of means of scale variables (e.g. age) amongst 25(OH)D quartiles. The hazard ratios (HR) are presented with 95% confidence interval (CI). Stepwise Cox multivariable proportional hazards regression models with total death, cardiac death and SCD as the dependent variable, and 25(OH)D and other variables as potential independent predictors (listed below) were fitted. To examine the differences in prognosis between subjects in the upper quartiles versus the lowest quartile of 25(OH)D, we adjusted for gender, age, smoking, hypertension, index diagnosis, DM, CHF (defined as Killip-Kimball class at admission; patients in class 2 to 4 were classified as CHF patients and patients in class 1 were classified as non CHF), history of previous CHD (i.e. history of either angina pectoris, MI, CABG, or PCI), hypercholesterolemia/use of statins, TnT>0.01 ng/mL, eGFR, hsCRP, BNP, BMI, months of the year and beta-blockers prior to enrolment. The Kaplan-Meier product limits were used for plotting the times to event with the equality of the 25(OH)D quartile survival curves assessed by the log-rank test.

In the discriminate analysis 25(OH)D and its natural logarithm were used as individual variables. The statistical analyses were performed using the statistical package SPSS version 19.0. All tests were two-sided with a significance level of 5%.

## Results

A total of 982 patients (588 men and 394 women) were enrolled in the ARRA-RACS study. Two samples were not available and four patients were lost to follow up. Thus 976 patients were left for the analysis of 25(OH)D as a predictor of outcome in our chest pain population. At index hospitalization, 388 patients (39.6%) had a peak TnT concentration exceeding 0.01 ng/mL. Mean age in the total population was 62.2±13.4 years.

The baseline characteristics, according to 25(OH)D quartiles at admission in all patients and in patients with TnT release, are shown in Table 1 and 2, respectively.

**Table 1:** Baseline characteristics of the total population arranged according to the quartiles of 25(OH)D.

	Quartiles of 25(OH)D				
Characteristics n (%)	Q1	Q2	Q3	Q4	p-value
25(OH)D (nmol/L)*	30.7±5.6	44.6±3.7	56.3±3.4	72.9±11.1	<0.0001
Age, years*	68.6±12.5	62.8±12.2	59.3±13.7	57.9±12.6	<0.0001
Male, n (%)	106 (43.3)	138 (56.6)	171 (69.5)	172 (70.2)	<0.0001
Smoking status, n (%)					0.383
Current Smoker, n (%)	53 (22.2)	61 (25.7)	62 (25.5)	61 (25.3)	
Past smoker, n (%)	148 (61.9)	132 (55.7)	126 (51.9)	130 (53.9)	
Never Smoked, n (%)	38 (15.9)	44 (18.6)	55 (22.6)	50 (20.7)	
Angina Pectoris, n (%)	46 (18.8)	61 (25.0)	68 (27.6)	48 (19.6)	0.054
CHF, n (%)					
Killip Class 2–4	59 (24.1)	33 (13.5)	29 (11.8)	44 (18.0)	0.001
History of previous MI, n (%)	28 (11.4)	23(9.4)	23 (9.3)	20 (8.2)	0.670
CABG, n (%)	9 (3.7)	17 (7.1)	12 (4.9)	9 (3.7)	0.275
PCI, n (%)	25 (10.2)	20 (8.2)	28 (11.4)	25 (10.2)	0.699
Hypertension, n (%)	178 (72.7)	159 (65.2)	143 (58.1)	153 (62.4)	0.007
History of DM 1, n (%)	7 (2.9)	6 (2.5)	2 (0.8)	0 (0.0)	0.029
History of DM 2, n (%)	72 (30.0)	49 (20.3)	38 (15.6)	28 (11.6)	<0.0001
STEMI, n (%)	34 (14.2)	35 (14.8)	29 (11.9)	46 (18.9)	0.181
TnT release, n (%)	116 (47.3)	89 (36.5)	90 (36.7)	93 (38.0)	0.041
eGFR (µmol L^−1^)*	77.8±32.5	82.7±29.4	84.6±25.9	80.0±25.1	0.041
Cholesterol/Statin, n (%)	41 (16.7)	40 (16.4)	39 (15.9)	39 (15.9)	0.993
Beta-blocker, n (%)	59 (24.5)	69 (28.6)	63 (25.9)	62 (25.7)	0.767
Known CHD, n (%)	71 (29.3)	89 (36.8)	91(37.1)	73 (29.9)	0.116
BMI (kg/m^2^)*	28.2±5.0	27.8±5.0	28.6±4.0	27.8±3.6	0.202
BNP Quartiles					<0.0001
Q1	52 (21.2)	53 (21.7)	73 (29.7)	67 (27.3)	
Q2	46 (18.8)	65 (26.6)	62 (25.2)	72 (29.4)	
Q3	54 (22.0)	66 (27.0)	66 (26.8)	59 (24.1)	
Q4	93 (38.0)	60 (24.6)	45 (18.3)	47 (19.2)	
hsCRP Quartiles					0.001
Q1	42 (17.3)	61 (25.1)	65 (26.4)	76 (31.0)	
Q2	58 (23.9)	55 (22.6)	73 (29.7)	61 (24.9)	
Q3	59 (24.3)	73 (30.0)	56 (22.8)	56 (22.9)	
Q4	84 (34.6)	54 (22.2)	52 (21.1)	52 (21.2)	

* Mean ± SD.

SD, standard deviation; 25(OH)D, 25-hydroxyvitamin D; CHF, congestive heart failure; MI, myocardial infarction; CABG, coronary artery bypass grafting; PCI, percutaneous coronary intervention; DM, diabetes mellitus; STEMI, ST-elevation myocardial infarction; TnT, troponin T; eGFR, estimated glomerular filtration rate; CHD, coronary heart disease; BMI, body mass index; BNP, B-type natriuretic peptide; hsCRP, high sensitivity C-reactive protein.

**Table 2:** Baseline characteristics of patients with a TnT release arranged according to the quartiles of 25(OH)D.

	Quartiles of 25(OH)D				
Characteristics n (%)	Q1	Q2	Q3	Q4	p-value
25(OH)D (nmol/L)*	28.6±5.4	42.3±3.9	55.3±3.6	73.1±14.0	<0.0001
Age, years*	70.0±12.5	65.7±11.5	63.2±10.8	59.2±13.7	<0.0001
Male, n (%)	48 (49.5)	58 (59.8)	77 (79.4)	75 (77.3)	<0.0001
Smoking status, n (%)					0.647
Current Smoker, n (%)	26 (27.4)	28 (29.8)	22 (22.7)	26 (27.1)	
Past smoker, n (%)	51 (53.7)	51 (54.3)	49 (50.5)	48 (50.0)	
Never Smoked, n (%)	18 (18.9)	15 (16.0)	26 (26.8)	22 (22.9)	
Angina Pectoris, n (%)	15 (15.5)	25 (25.8)	29 (29.9)	25 (25.8)	0.111
CHF, n (%)					
Killip Class 2–4	29 (29.9)	21 (21.6)	13 (13.4)	22 (22.7)	0.051
History of previous MI, n (%)	13 (13.4)	15 (15.5)	10 (10.3)	10 (10.3)	0.634
CABG, n (%)	3 (3.1)	9 (9.6)	9 (9.3)	4 (4.2)	0.145
PCI, n (%)	9 (9.3)	12 (12.4)	16 (16.5)	10 (10.3)	0.426
Hypertension, n (%)	70 (72.2)	63 (64.9)	54 (55.7)	59 (60.8)	0.107
History of DM 1, n (%)	6 (6.2)	2 (2.2)	1 (1.0)	0 (0.0)	0.026
History of DM 2, n (%)	39 (40.2)	25 (26.9)	22 (22.7)	13 (13.5)	0.0003
STEMI, n (%)	27 (27.8)	27 (29.7)	24 (24.7)	40 (41.7)	0.060
eGFR (µmol L^−1^)*	72.1±36.0	73.8±32.3	82.1±32.9	73.2±29.1	0.126
Cholesterol/Statin, n (%)	18 (18.6)	21 (21.6)	19 (19.6)	15 (15.5)	0.737
Beta-blocker, n (%)	21 (21.6)	23 (24.5)	31 (32.3)	24 (25.5)	0.383
Known CHD, n (%)	24 (24.7)	40 (41.7)	40 (41.2)	36 (37.1)	0.049
BMI (kg/m^2^)*	27.6±4.8	27.3±4.7	27.9±3.9	27.7±3.2	0.810
BNP Quartiles					0.004
Q1	19 (19.6)	17 (17.5)	36 (37.1)	25 (25.8)	
Q2	16 (16.5)	28 (28.9)	22 (22.7)	31 (32.0)	
Q3	28 (28.9)	25 (25.8)	19 (19.6)	25 (25.8)	
Q4	34 (35.1)	27 (27.8)	20 (20.6)	16 (16.5)	
hsCRP Quartiles					0.115
Q1	19 (20.0)	21 (21.9)	27 (27.8)	30 (30.9)	
Q2	22 (23.2)	23 (24.0)	25 (25.8)	24 (24.7)	
Q3	20 (21.1)	25 (26.0)	30 (30.9)	23 (23.7)	
Q4	34 (35.8)	27 (28.1)	15 (15.5)	20 (20.6)	

* Mean ± SD.

SD, standard deviation; 25(OH)D, 25-hydroxyvitamin D; CHF, congestive heart failure; MI, myocardial infarction; CABG, coronary artery bypass grafting; PCI, percutaneous coronary intervention; DM, diabetes mellitus; STEMI, ST-elevation myocardial infarction; TnT, troponin T; eGFR, estimated glomerular filtration rate; CHD, coronary heart disease; BMI, body mass index; BNP, B-type natriuretic peptide; hsCRP, high sensitivity C-reactive protein.

In the total population there was a significant and gradual decrease in age from the lowest (Q1) to the highest quartile (Q4) of 25(OH)D and a higher proportion of patients with a TnT exceeding 0.01 ng/mL was noted in Q1 as compared to higher quartiles. In addition, more patients with hypertension, DM type 1 and 2 and heart failure, were found in Q1. The lowest quartile also had higher levels of BNP, hsCRP and lower levels of eGFR. Similar differences (except for hypertension, eGFR and hsCRP) were found in patients with a TnT release. Moreover, in the latter subpopulation more CHD patients were found in the higher quartiles as compared to Q1.

**Figure 1:**
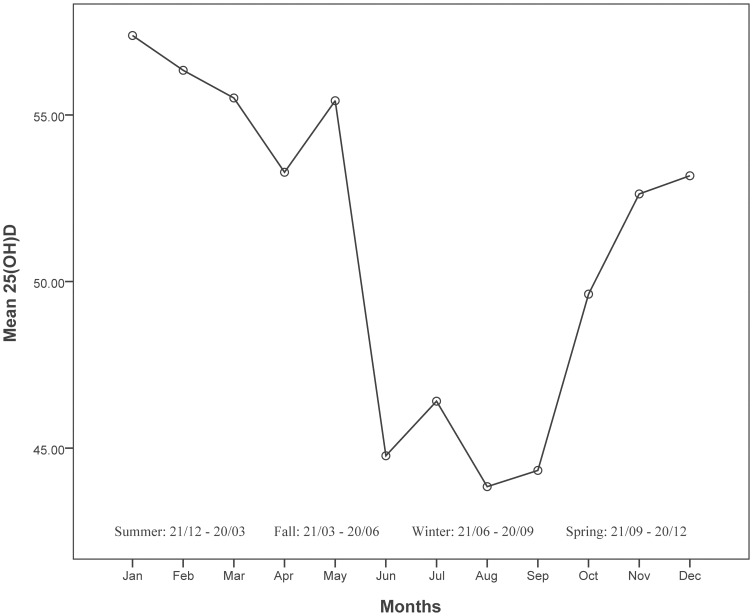
Mean 25(OH)D levels by month.

The variations in 25(OH)D by month are shown in Figure 1 and significant seasonal changes in serum 25(OH)D levels were found. The mean 25(OH)D value was 54.1 nmol/L (95% CI, 52.6 nmol/L–55.5 nmol/L) between October and March, and 48.3 nmol/L (95%CI, 46.9 nmol/L–49.8 nmol/L) between April and September; p<0.0001.

A significantly higher proportion of patients dying was found in Q1 of 25(OH)D as compared to Q4, both in the total population (25.3% vs 6.1%) and in patients with a TnT release, (43.3% vs 9.3%) each p<0.0001, respectively.

25(OH)D status and log_e_ (25(OH)D) were used as individual variables in a discriminate analysis (Table 3) with the purpose to predict fatal outcome in the total population and in patient groups with and without TnT release, respectively. The specificity of non-logarithmic 25(OH)D for predicting all-cause mortality in the total population was 62.2%, with a sensitivity of 67.2%.

**Table 3:** Discriminate analysis of all cause mortality using 25(OH)D and their natural logarithm, for the total patient population and in patients groups with and without TnT release, respectively.

Population		25(OH)D	Log 25(OH)D
Total	Specificity	62.2	68.0
	Sensitivity	67.2	62.2
	Overall	62.8	67.3
	p-value	<0.0001	<0.0001
TnT release	Specificity	63.2	69.1
	Sensitivity	72.0	67.1
	Overall	65.0	68.7
	p-value	<0.0001	<0.0001
No TnT release	Specificity	55.8	63.2
	Sensitivity	59.5	56.8
	Overall	56.0	62.8
	p-value	0.018	0.010

25(OH)D, 25-hydroxyvitamin D; TnT, troponin T.

### Total Patient Population

#### All-cause mortality, cardiac death and sudden cardiac death

**Figure 2:**
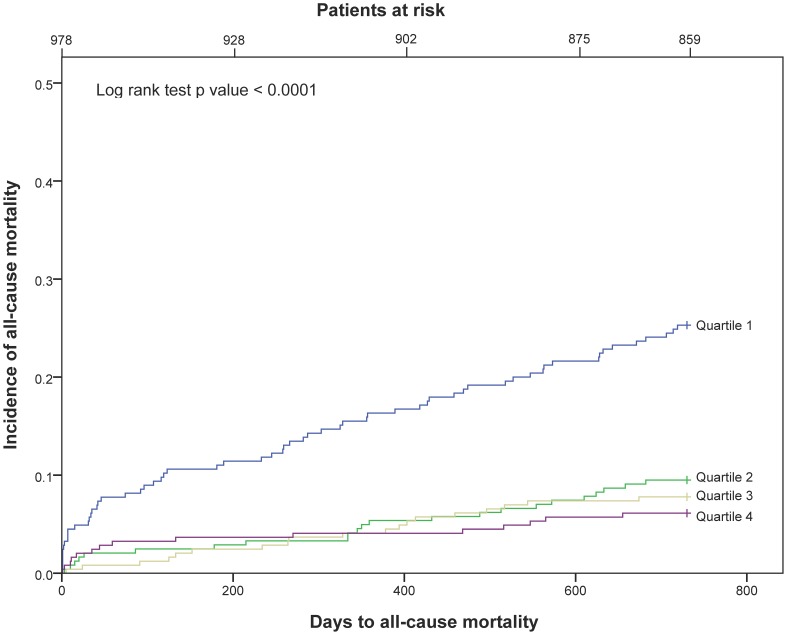
Kaplan-Meier plots for total mortality of 25(OH)D quartiles in the total patient population.

After a follow-up period of 24 months, 119 patients (12.2%) had died. In 66 patients (6.9%) death was defined as cardiac, of which 50 patients (5.3%) were characterized as SCD. Kaplan-Meier survival curves for all-cause mortality in the 25(OH)D quartiles at baseline for all patients are presented in Figure 2.

**Figure 3:**
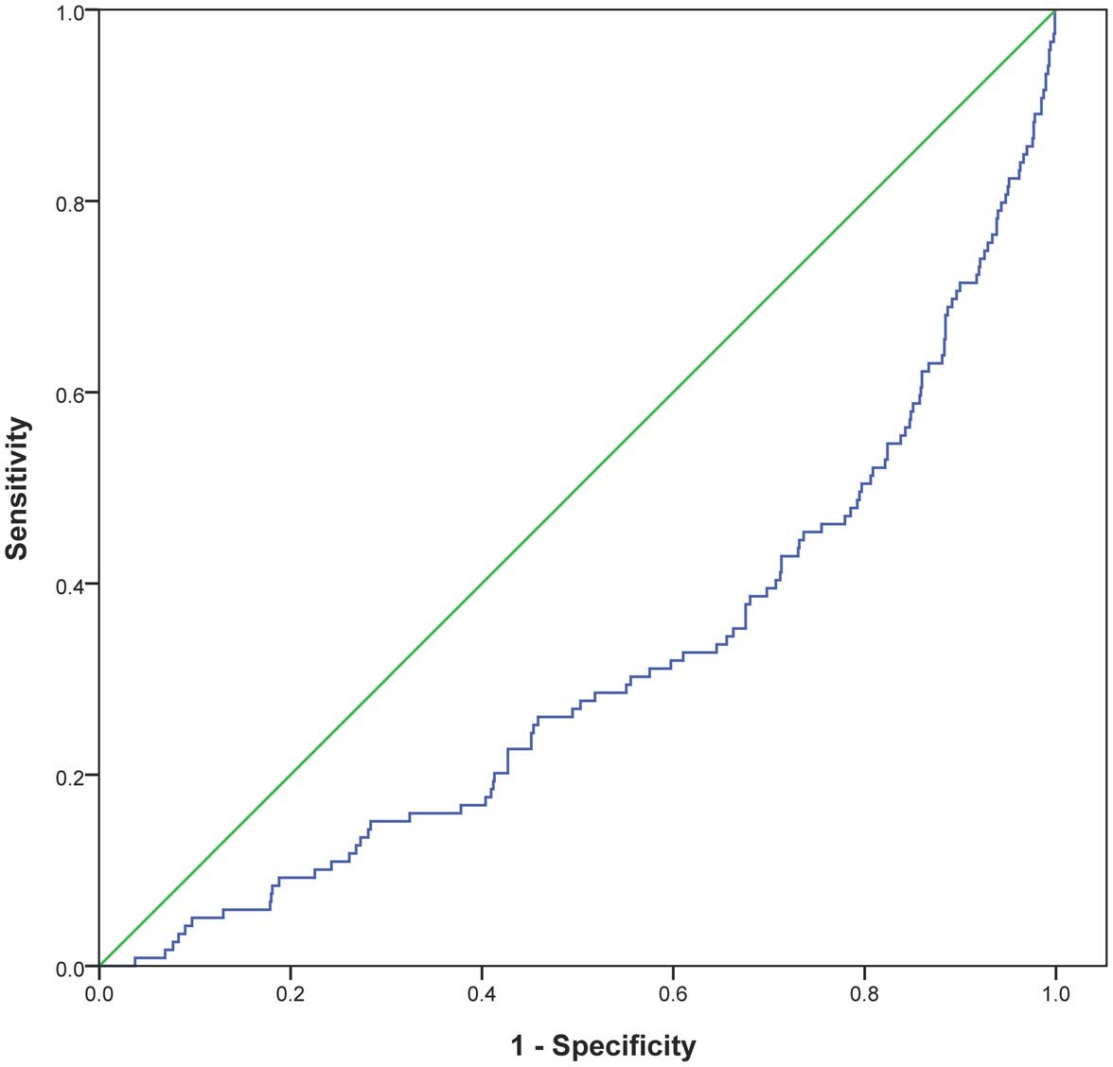
Receiver operated characteristics curve for 25(OH)D related to all-cause mortality in the total patient population.

Receiver operated characteristics (ROC) curve for 25(OH)D for all-cause mortality is shown in Figure 3. The area under the ROC for 25(OH)D was 0.307 (p<0.0001).

In the univariate Cox regression analysis there was a statistically highly significant difference between each of the upper three quartiles' HR as compared to Q1 of 25(OH)D in the total patient population with respect to all-cause mortality, cardiac death and SCD.

When comparing 25(OH)D in Q4 to Q1 in a multivariable Cox regression model for all-cause mortality within 2 years in the total patient population, the HR was 0.37 (95% CI, 0.19–0.73), p = 0.004. For cardiac death, the HR was 0.23 (95% CI, 0.08–0.67), p = 0.007, and for SCD the HR was 0.32 (95% CI, 0.11–0.94), p = 0.038 (Table 4).

**Table 4:** Hazard ratios for 25(OH)D, age, TnT, BNP, hsCRP and BMI.

	Total patient population		
Variables	All-cause mortality	Cardiac Death	Sudden Cardiac Death
25(OH)D	0.37 (0.19–0.73)	0.23 (0.08–0.67)	0.32 (0.11–0.94)
Age	1.06 (1.04–1.08)	1.05 (1.03–1.08)	1.08 (1.05–1.12)
TnT	2.58 (1.67–4.00)	2.33 (1.29–4.20)	3.17 (1.61–6.22)
BNP	1.97 (1.02–3.81)	2.80 (1.03–7.60)	NA
hsCRP	1.76 (1.00–3.11)	NA	NA
BMI	NA	NA	1.07 (1.00–1.13)

Values are given as hazard ratio (95% CI).

25(OH)D, 25-hydroxyvitamin D; TnT, troponin T; BNP, B-type natriuretic peptide; hsCRP, high sensitivity C-reactive protein; BMI, body mass index.

### Patients with troponin T release

#### All-cause mortality, cardiac death and sudden cardiac death

**Figure 4:**
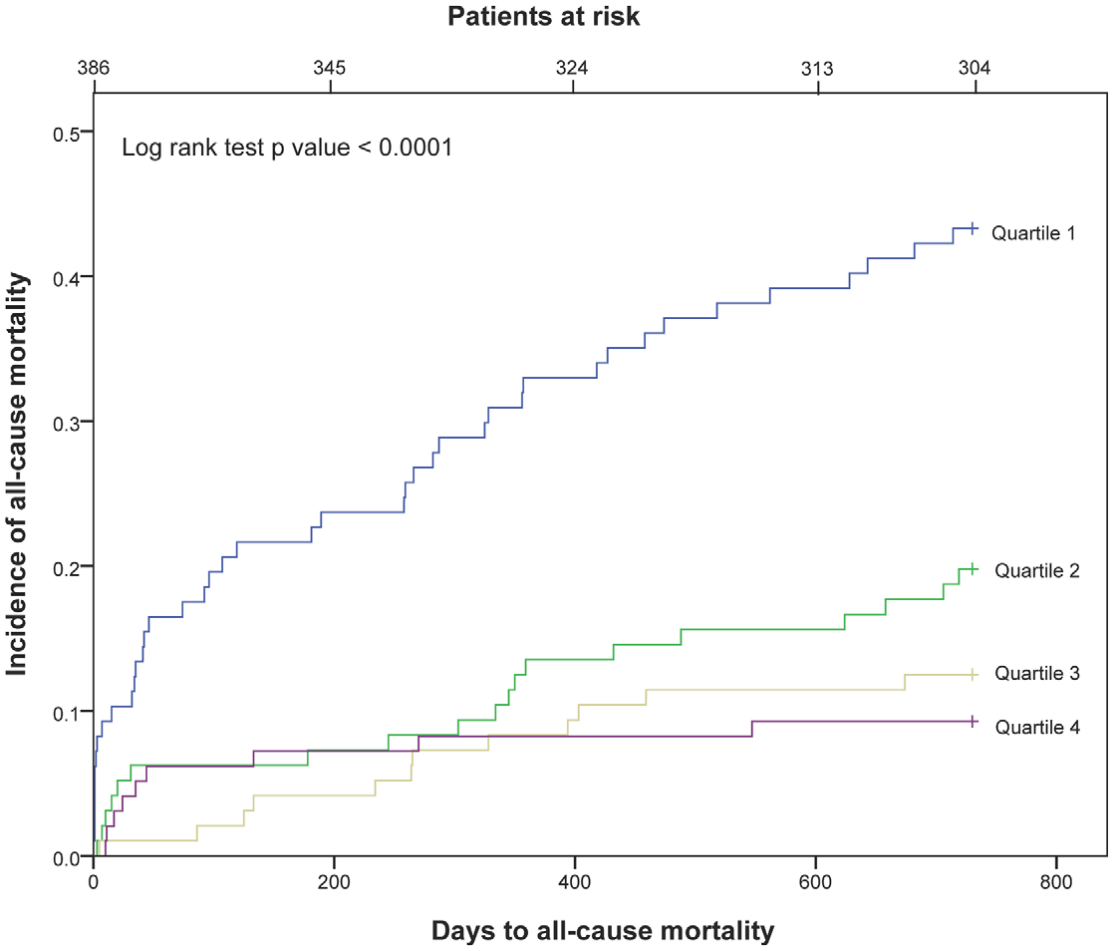
Kaplan-Meier plots for total mortality of 25(OH)D quartiles in patients with TnT release.

In the 388 patients admitted with TnT release we found that 82 patients (21.2%) had died during the 2-year follow-up. Death was categorized as cardiac in 44 patients (12.1%), of whom 35 deaths (9.5%) were defined as sudden. Kaplan-Meier curves for the cumulative risk of total mortality in the 25(OH)D quartiles for patients with a TnT release are presented in Figure 4.

**Figure 5:**
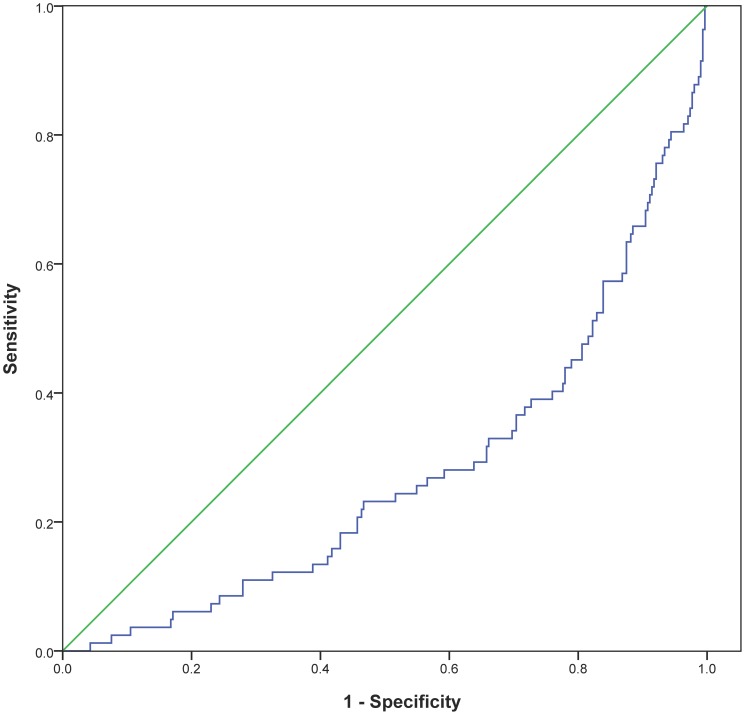
Receiver operated characteristics curve for 25(OH)D related to all-cause mortality in patients with TnT release.

ROC curve for 25(OH)D in patients with a TnT release is shown in Figure 5. The area under the ROC curve for 25(OH)D was 0.276 (p<0.0001).

There was a statistically highly significant difference between each of the upper three quartiles as compared to Q1 of 25(OH)D in the univariate analysis of all cause mortality, cardiac death and SCD, respectively.

When comparing 25(OH)D in Q4 to Q1 in a multivariable Cox regression model for all-cause mortality within 2 years in patients with TnT release, the HR was 0.24 (95% CI, 0.10–0.54), p = 0.001. For cardiac death, the HR was 0.18 (95% CI, 0.05–0.60), p = 0.006, and for SCD, the HR was 0.25 (95% CI, 0.07–0.89), p = 0.033 (Table 5).

**Table 5:** Hazard ratios for 25(OH)D, age, TnT, BNP, hsCRP and BMI.

	Patients with TnT release		
Variables	All-cause mortality	Cardiac Death	Sudden Cardiac Death
25(OH)D	0.24 (0.10–0.54)	0.18 (0.05–0.60)	0.25 (0.07–0.89)
Age	1.06 (1.03–1.08)	1.07 (1.03–1.10)	1.07 (1.03–1.11)
TnT	NA	NA	NA
BNP	NA	NA	NA
hsCRP	3.32 (1.68–6.59)	NA	NA
BMI	NA	NA	NA

Values are given as hazard ratio (95% CI).

25(OH)D, 25-hydroxyvitamin D; TnT, troponin T; BNP, B-type natriuretic peptide; hsCRP, high sensitivity C-reactive protein; BMI, body mass index.

### Patients without troponin T release

#### All-cause mortality, cardiac death and sudden cardiac death

After a follow-up period of 24 months, 37 patients (6.3%) of 593 with no TnT release had died. In the univariate analysis of all-cause mortality in these patients, the HR for 25(OH)D was 0.39 (95% CI, 0.15–1.00), p = 0.05, whereas 25(OH)D status did not add any prognostic information related to cardiac death and SCD.

## Discussion

This prospective observational study was designed to evaluate the prognostic utility of 25(OH)D in admission samples from consecutively included chest pain patients with suspected ACS in a beef-eating population living at a high altitude in a subtropical inland city of Argentina. We performed a comparative interquartile analysis of 25(OH)D as a prognostic biomarker in the total population, and in patients with and without TnT release, respectively.

We have previously confirmed the prognostic value of the established risk markers BNP and hsCRP in this study population [19], and in the multivariable analysis of 25(OH)D as a prognostic indicator we have corrected for these predictors in order to evaluate its prognostic independency.

After correcting for other possible confounders including cardiovascular risk factors, the use of medications, age, gender, BMI and the sampling time, we were able to demonstrate a statistically significant association between low levels of 25(OH)D and 2 year survival for total mortality, cardiac death and SCD, both in the total population and in patients with a TnT release. Several other markers in addition to 25(OH)D were also found to predict future mortality, as shown in Tables 4 and 5. Due to the geographical location close to the Equator and less seasonal variations as compared to tempered zones, we chose to test for month of sampling as a potential confounder instead of seasons. However, applying seasons in our model made no difference.

Several other observational and epidemiological studies have also shown an inverse association between both all-cause and cardiac mortality and vitamin D. In the NHANSE III study, the lowest 25(OH)D quartile was associated with a higher risk of all-cause mortality in the general US population [10] as well as in older US adults (age >65) [12]. In the general US population, the CVD mortality showed a similar trend, but did not remain statistically significant in the fully adjusted model, whereas CVD mortality was found to be statistically significant in the older US population. The Tromsø study [9] showed a significantly increased risk of all-cause death in the lowest 25(OH)D quartile as compared to the highest in the non-smoking population, whereas 25(OH)D did not predict CVD outcome. Furthermore, low levels of 25(OH)D were associated with all-cause and cardiovascular mortality in the LURIC study [7], which included clinically stable patients referred for coronary angiography. In that population, patients with low levels of 25(OH)D were found to have a higher mortality rate due to heart failure and SCD [15]. The Framingham Offspring Study [6] demonstrated that low levels of 25(OH)D were associated with higher CVD risk in patients with hypertension. In the Nurses' Health Study and the Health Professionals Follow-Up Study [8] it was suggested that a higher vitamin D intake correlated with a lower risk of CVD in men but not in women. In the Mini-Finland Health Survey, Kilkkinen et al. [5] demonstrated that a low level of 25(OH)D may be associated with a higher risk of a fatal CVD event. Two other studies suggest a higher prevalence of 25(OH)D deficiency in patients with acute MI [13]–[14].

In our study, patients were recruited from a subtropical area at an altitude above 1000 m and included an Hispanic population. Despite the geographical location there were significant seasonal changes in 25(OH)D levels. The contribution of diet to vitamin D status is probably negligible in this population as the intake of fatty fish, the primary source of vitamin D in the diet, is very low. Also, the food fortification with vitamin D in Argentina was introduced at the end of 2010.

Despite the availability of vitamin D through sun exposure, a high proportion of this population demonstrated subnormal levels of 25(OH)D, which could be explained by their lifestyle with long working hours and a siesta in the middle of the day, avoiding the sun due to the heat. Furthermore, the majority of this population belonged to an urban and sheltered environment. Although these patients were living at a high altitude, this altitude would not promote noteworthy additional exposure to UV radiation.

Patients in Q1 of 25(OH)D would be classified as vitamin D deficient according to the general agreement. In this quartile, patients were older, included more females, had higher rates of DM, heart failure, hypertension, lower eGFR, and a higher proportion presented with a TnT release. The lowest quartile also had higher levels of BNP, hsCRP and less CHD patients. Except for hypertension and eGFR, similar differences were found in patients with a TnT release, in which the highest quartile also included more STEMI, with a borderline significant p-value of 0.06.

In both univariate and multivariate analysis, we demonstrated a statistically highly significant increase in all-cause mortality, cardiac and SCD in Q1 as compared to Q4, both in the total patient population and in patient with a TnT release. After adjusting for covariates, the prognostic utility of 25(OH)D was maintained for all end-points.

ROC analysis supports our results related to low 25(OH)D values and high mortality in the total population (0.307, p<0.0001) and in the population with TnT release (0.276, p<0.0001). Although the AUC score in the ROC analysis is low, the low scores reflect high mortality, as it represents the lower and not the higher quartile of 25(OH)D values. The opposite relation would yield a value in the upper half of the ROC.

A strength of this study would be the inclusion of patients with suspected ACS, collection of blood samples at admission, a planned sub-group analysis according to TnT release and a prospective design, evaluating the prognostic value of 25(OH)D in relation to pre-specified endpoints consisting of total mortality, cardiac and sudden cardiac death. Furthermore, the study patients were regularly contacted and only two patients were lost to follow up. Treatment was based on strict adherence to ACC recommendations [23] at all hospitals and included aspirin, clopidogrel and statins as secondary preventions and specific management related to revascularization (usually limited to primary PCI) and to complications such as arrhythmias and heart insufficiency [19].

Some limitations of this study merit consideration. Concentrations of 25(OH)D in the healthy state prior to hospitalization remain unknown and our analyses are based on a single baseline measurement. As patients in our ACS registry were strictly treated according to ACC guidelines, medication was not recorded specifically post discharge.

Differentiation of chest pain was usually performed prior to hospitalization and our patients were included after admission with a suspected ACS diagnosis. As this is not a randomized study but a registry with few exclusion criteria limited to age, consent and prior inclusion, all ACS patients who were asked to participate, accepted the invitation, except for two subjects. We cannot provide the exact number of patients who were not asked. However, in the private hospitals in which 94.5% of the study population was included, the admission rate was lower than in the public hospital, and these patients were generally accounted for.

Although we did not adjust for left ventricular ejection fraction, we did adjust for BNP and known CHF (Killip-Kimball class). Furthermore, we did not correct for parathyroid hormone.

According to our results, 25(OH)D may serve as a useful predictor of all-cause and cardiac mortality including SCD. Randomized controlled trials are needed to evaluate whether ACS patients may profit therapeutically by vitamin D intervention.
